# Impaired Dynamic Cerebral Autoregulation Across the REM Sleep Behavior Disorder Spectrum: Evidence From Isolated REM Sleep Without Atonia to Parkinson's Disease With RBD

**DOI:** 10.1002/cns.71190

**Published:** 2026-10-21

**Authors:** Yanan Zhang, Ying Piao, Qingqing Sun, Huimin Li, Yaru Wang, Jun Lu, Zhenni Guo, Zan Wang

**Affiliations:** ^1^ Sleep Center, Department of Neurology The First Hospital of Jilin University Changchun China; ^2^ Stroke Center, Department of Neurology The First Hospital of Jilin University Changchun China

**Keywords:** autonomic nerve dysfunction, dynamic cerebral autoregulation, isolated rapid eye movement sleep behavior disorder, isolated rapid eye movements sleep without atonia

## Abstract

**Objectives:**

Rapid eye movement (REM) sleep behavior disorder (RBD) is a parasomnia characterized by REM sleep without atonia (RSWA) and dream‐enactment behaviors and is recognized as a prodromal manifestation of α‐synucleinopathies. Isolated RSWA (iRSWA) has been proposed as a potential early stage within the RBD spectrum, although its clinical and physiological significance remains incompletely understood. This study aimed to investigate alterations in sleep architecture, phasic muscle activity, and dynamic cerebral autoregulation (dCA) across the spectrum from iRSWA to isolated RBD (iRBD) and Parkinson's disease with RBD (PD + RBD).

**Methods:**

We conducted a case–control study involving patients with iRSWA, iRBD, and PD + RBD, as well as age‐ and sex‐matched healthy controls, based on video‐polysomnography and magnetic resonance imaging. Scales for Outcomes in Parkinson's Disease‐Autonomic Dysfunction (SCOPA‐AUT) were used to evaluate the autonomic function. Transcranial Doppler and finger plethysmography were used to assess dCA by analyzing the phase difference using transfer function analysis.

**Results:**

A total of 25 patients with iRSWA, 30 with iRBD, 28 with PD + RBD, and 30 healthy controls were included. REM sleep percentage was significantly lower in the iRBD and PD + RBD groups than in controls. Phasic RSWA, but not tonic RSWA, differed significantly among the three patient groups (*p* < 0.001). Phase difference was significantly lower in all three patient groups than in controls (*p* < 0.001) and was significantly associated with REM sleep amount and phasic RSWA (both *p* < 0.001).

**Conclusions:**

These findings suggest that dCA is impaired in patients with iRSWA, iRBD, and PD + RBD. Reduced phase difference was associated with lower REM sleep amount and greater phasic RSWA, suggesting disrupted coupling among REM sleep regulation, autonomic function, and cerebrovascular control. dCA dysfunction may represent an early pathophysiological feature in RBD‐related neurodegeneration.

**Trial Registration:**

ClinicalTrials.gov Identifier: NCT02752139

## Graphical abstract

The progression from isolated REM sleep without atonia (iRSWA) to isolated REM sleep behavior disorder (iRBD) and Parkinson's disease with RBD, accompanied by increasing autonomic dysfunction and REM sleep disruption. Elevated phasic RSWA, reduced REM sleep duration, autonomic dysfunction, and neurotransmitter dysregulation contribute to impaired dynamic cerebral autoregulation.

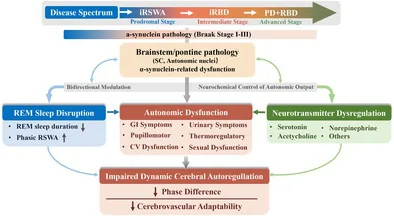

## 1 Introduction

Rapid eye movement (REM) sleep behavior disorder (RBD) is a parasomnia characterized by REM sleep without atonia (RSWA) and recurrent nocturnal dream‐enactment behavior, often accompanied by vivid, violent dreams and injury to oneself and the bedpartner, which may be isolated or associated with neurodegenerative diseases [1]. The prevalence of RBD in the general population ranges from 0.38% to 2.01% but is substantially higher among individuals with neurodegenerative diseases, particularly α‐synucleinopathies [2].

RSWA detected by polysomnography (PSG) is the core objective hallmark of RBD. Isolated RSWA (iRSWA), defined as RSWA occurring in the absence of dream‐enactment behaviors or an identifiable underlying cause, has been proposed as a potential prodromal stage within the RBD spectrum [3]. Longitudinal cohort studies have shown that 26.8% of individuals with iRSWA progressed to isolated RBD (iRBD), whereas 8.9% developed neurodegenerative diseases during a 4‐year follow‐up period [4]. Moreover, iRBD is now widely recognized as one of the earliest clinical markers of α‐synuclein‐related neurodegeneration, including Parkinson's disease (PD) and dementia with Lewy bodies (DLB) [5]. The estimated risk of phenoconversion to a clinically manifest neurodegenerative syndrome is 17.7%–33.1% at 5 years, 40.9%–75.7% at 10 years, and as high as 90.9% at 14 years [6, 7, 8].

Several studies have shown that patients with RBD frequently exhibit autonomic dysfunction, most commonly manifested as orthostatic hypotension, reduced heart rate variability, gastrointestinal disturbances, and urinary symptoms [9, 10]. In a multicenter cohort of 340 participants with iRBD, 27% met the criteria for orthostatic hypotension (OH). Among those with OH, 77% exhibited an attenuated heart rate response, suggesting concomitant cardiovascular autonomic dysfunction, and 72% had coexisting supine hypertension [10]. Such autonomic abnormalities may also affect dynamic cerebral autoregulation (dCA), an intrinsic regulatory mechanism that stabilizes cerebral blood flow in response to fluctuations in systemic blood pressure. dCA reflects the instantaneous adjustments in cerebral blood flow in response to rapid and substantial fluctuations in arterial blood pressure, reflecting the instantaneous rate of change of cerebral blood flow to cerebral perfusion pressure [11]. This process is mediated by a complex interplay of endothelial, myogenic, metabolic, and neurogenic mechanisms [12], with the autonomic nervous system contributing importantly to its neurogenic regulation [13]. During REM sleep, autonomic activity becomes highly variable and is modulated by brainstem REM sleep‐generating circuits [14, 15].

Given the frequent autonomic dysfunction observed in RBD, dCA may also be affected, potentially increasing vulnerability to cerebrovascular and neurodegenerative processes [16]. Consistent with this possibility, our previous study demonstrated impaired dCA in patients with iRBD [17]. However, dCA has rarely been investigated in individuals with iRSWA, and direct comparisons across iRSWA, iRBD, and PD with RBD (PD + RBD) remain lacking.

This study aimed to assess dCA across healthy controls and patients with iRSWA, RBD, and PD + RBD, and to investigate the associations between dCA parameters and the severity of REM sleep dysregulation.

## 2 Methods

### 2.1 Subjects

A case–control study was conducted among patients attending the outpatient clinic of the Department of Neurology, the First Hospital of Jilin University, between March 2021 and December 2023. The study protocol was approved by the Ethics Committee of the First Hospital of Jilin University. All patients who underwent overnight video‐polysomnography (v‐PSG) during the study period were screened for eligibility. The final study population included individuals diagnosed with iRSWA, iRBD, and PD + RBD, as confirmed by a neurologist specializing in sleep disorders. The main indications for v‐PSG were: (1) excessive daytime sleepiness and/or fatigue; (2) suspected sleep‐related breathing disorders; (3) difficulty initiating or maintaining sleep; (4) suspected sleep‐related movement disorders; (5) a combination of two or more of the above conditions; and (6) other sleep‐related complaints that did not fit the previous categories. Participants were not recruited specifically for this study but were retrospectively identified from a broader sleep clinic population who underwent v‐PSG for clinical indications or participation in other research protocols.

The presence of iRSWA was defined according to the American Academy of Sleep Medicine (AASM) scoring criteria. The diagnosis of iRBD was based on the International Classification of Sleep Disorders, Third Edition [18], and PD + RBD was diagnosed according to the United Kingdom Parkinson's Disease Society Brain Bank criteria [19]. The inclusion criteria for each group were as follows: (1) iRSWA group: RSWA documented by v‐PSG without reported dream‐enactment behavior, an RBD questionnaire‐Hong Kong (RBDQ‐HK) score < 18, and no diagnosis of PD, dementia, multiple system atrophy, or other neurodegenerative disorders; (2) iRBD group: recurrent sleep‐related vocalizations and/or complex motor behaviors documented by v‐PSG or explicit dream interpretation during REM; evidence of RSWA on v‐PSG, and no diagnosis of PD, dementia, multiple system atrophy, or other neurodegenerative disorders; (3) PD + RBD group: patients meeting the diagnostic criteria for both RBD and PD according to the United Kingdom Parkinson's Disease Society Brain Bank criteria; and (4) control group: age‐ and sex‐matched healthy volunteers without sleep‐related complaints.

The exclusion criteria were as follows: (1) current or recent use of antidepressant or antipsychotic medications within the preceding 3 months; (2) moderate‐to‐severe obstructive sleep apnea [apnea–hypopnea index (AHI) ≥ 15 events/h], narcolepsy, a history of non‐REM (NREM) sleep parasomnia (including confusional arousals, sleepwalking, or sleep terrors), or other neurological or psychiatric disorders; (3) arrhythmia, hyperthyroidism, or other conditions potentially affecting systemic hemodynamics; and (4) intracranial or extracranial vascular stenosis or occlusion identified by vascular ultrasonography.

Demographic and clinical characteristics, including age, sex, marital status, and body mass index (BMI), were collected for all participants.

### 2.2 Sample Size Estimation

Sample size estimation was based on the primary outcomes of REM sleep amount, phasic RSWA, and dCA‐derived phase difference. Previous studies in RBD‐related populations have reported substantial between‐group differences in REM sleep parameters, quantitative RSWA measures, and cerebral autoregulation indices compared with healthy controls, supporting the assumption of a large effect size (Cohen's *f* > 0.40) [20, 21, 22]. Based on a one‐way analysis of variance with four groups, a two‐sided significance level of 0.05 and a statistical power of 0.80, the minimum required sample size was estimated to be approximately 20 participants per group.

### 2.3 Questionnaires

The RBDQ‐HK is a 13‐item self‐report questionnaire designed for both screening and assessing symptom severity in patients with RBD. It evaluates clinical manifestations and symptom severity by assessing both lifetime occurrence and the frequency of RBD‐related symptoms over the preceding year, with a total score ranging from 0 to 100. The questionnaire comprises two main domains reflecting dream‐related and behavioral manifestations. Previous studies have shown that a cutoff score of 18/19 provides a sensitivity of 82.2% and a specificity of 86.9% for identifying probable RBD [23]. The RBDQ‐HK has therefore been widely utilized in clinical and research settings for both RBD screening and symptom assessment [24].

Autonomic function was assessed using the Scales for Outcomes in Parkinson's Disease‐Autonomic Dysfunction (SCOPA‐AUT), a widely validated and practical instrument for evaluating autonomic dysfunction [25]. The SCOPA‐AUT consists of 25 items covering six domains: gastrointestinal (7 items), urinary (6 items), cardiovascular (3 items), thermoregulatory (4 items), pupillomotor (1 item), and sexual function (2 items). Each item is scored 0 (never), 1 (sometimes), 2 (regularly), or 3 (often), with a maximum total score of 69. A domain was considered impaired when the corresponding domain score was ≥ 1, indicating the presence of at least one symptom occurring at least occasionally.

### 2.4 V‐PSG

All participants underwent overnight v‐PSG monitoring (Compumedics, Abbotsford, Australia) for a minimum duration of 8 h at the sleep center of our hospital. Before PSG recording, participants were instructed to avoid caffeinated beverages, alcohol, and sleep medicines. The v‐PSG montage included electroencephalography, electrooculography, chin electromyography (EMG), bilateral flexor digitorum superficialis electromyography, bilateral tibialis anterior electromyography, electrocardiography, nasal pressure, finger oximetry, chest and abdominal respiratory bands. PSG recordings were analyzed by certified sleep technicians with PSG technologist certification by referring to the revised interpretation criteria of sleep stages and related events issued by the AASM version 2.6. Parameters including the total sleep time (TST), sleep onset latency (SL), sleep efficiency (SE), REM sleep, REM sleep latency, Stage 1 NREM (stage N1), Stage 2 NREM (stage N2), Stage 3 NREM (Stage N3), arousal index, periodic limb movement index (PLMI), AHI, total RSWA, tonic RSWA, and phasic RSWA were calculated and recorded.

RSWA included sustained muscle activity (tonic activity) of the chin EMG and excessive transient muscle activity (phasic activity) of the chin or limb EMG during REM sleep according to the AASM scoring manual guidelines [26]. Tonic activity was defined as a 30‐s epoch of REM sleep with at least 50% of the duration of the epoch having chin EMG amplitude greater than the minimum amplitude demonstrated in NREM sleep. Phasic activity was defined as dividing the 30‐s epoch of REM sleep into sequential, 3‐s mini‐epochs, and at least 50% of the mini‐epochs (3 s) contain transient muscle activity in the chin or limb EMG, lasting 0.1–5.0 s in duration and at least 4 times as high in amplitude as the background EMG activity. All EMG activity due to respiratory events, snoring, arousals, or periodic limb movements was excluded from analysis. Patients with indefinable REM muscle tone due to severe EMG artifacts were also excluded. Tonic RSWA percentage (tonic RSWA %) and phasic RSWA percentage (phasic RSWA %) were calculated as the proportion of REM sleep epochs meeting the respective criteria and were used as quantitative measures of RSWA severity.

For the quantitative diagnosis of abnormal RSWA, a cutoff of ≥ 27% was applied using 30‐s REM sleep epochs, based on “any” EMG activity in the chin and/or phasic EMG activity in the bilateral flexor digitorum superficialis muscles [22].

### 2.5 Dynamic Cerebral Autoregulation Measurement and Analysis

All participants were instructed to abstain from caffeine, alcohol, nicotine, and strenuous physical activity for at least 12 h before the examination. All measurements were conducted in a dedicated, quiet laboratory. Baseline heart rate (HR) and arterial blood pressure were first measured using an automated sphygmomanometer (Omron 711; Omron, Kyoto, Japan). Subsequently, bilateral middle cerebral artery blood flow velocity was continuously recorded for 10 min using transcranial Doppler ultrasonography (MultiDop X2; DWL, Sipplingen, Germany), together with beat‐to‐beat arterial blood pressure measured by finger plethysmography (Finometer Model 1; Finapres Medical Systems, Enschede, Netherlands). End‐tidal carbon dioxide was continuously monitored using a capnograph connected to a facemask and nasal cannula. The recorded signals were stored for subsequent dCA analysis. All measurements were performed by an experienced operator to ensure consistency and data quality.

MATLAB software (MathWorks, Natick, MA) was used to process the recorded data. Transfer function analysis (TFA) was used to perform the dCA analysis. Frequency‐dependent phase difference was derived from the transfer function between arterial blood pressure and cerebral blood flow velocity, calculated as the ratio of their cross‐spectrum to the autospectrum of arterial blood pressure. Analyses were performed within the low‐frequency range of 0.06–0.12 Hz, which is considered particularly relevant to cerebral autoregulatory hemodynamics [27]. A lower phase difference was interpreted as indicating impaired dCA. The reliability of the frequency‐domain relationship between arterial blood pressure and cerebral blood flow velocity was evaluated using coherence, and only data segments with coherence > 0.5 were included in the statistical analysis.

### 2.6 Statistical Analysis

SPSS software (version 26.0; IBM, Armonk, NY) was used for statistical analysis. The Shapiro–Wilk test was used to assess the normality of continuous variables. Data with a normal distribution were expressed as mean and standard deviation, whereas data with skewed distribution were expressed as medians with interquartile range. Categorical data were expressed as absolute values and percentages. One‐way analysis of variance or the Kruskal–Wallis *H* test was used to compare differences between multiple groups of independent samples based on the data distribution. Chi‐squared test was used to compare differences between multiple groups of independent samples. Analysis of covariance was used to assess variables with confounding factors interference. The correlation between phase difference and phasic RSWA was examined using the Pearson correlation test. Univariate and multivariate linear regression were used to assess the association between phase difference and clinical parameters. In the post hoc analysis, the Bonferroni method was used to calculate the adjusted *p*‐value. Statistical significance was set at *p* < 0.05.

## 3 Results

### 3.1 Baseline Characteristics

Among the 135 participants who completed v‐PSG (Figure 1), 31 had iRSWA, 35 had iRBD, 37 had PD + RBD, and 32 were healthy controls. After application of the exclusion criteria, 25 participants with iRSWA, 30 with iRBD, 28 with PD + RBD, and 30 healthy controls remained for the final analysis. During v‐PSG, observable REM sleep‐related vocalizations and/or dream‐enactment behaviors were documented in 20 of 30 patients with iRBD (66.7%) and 22 of 28 patients with PD + RBD (78.6%). All variables were compared across the four groups (Table 1). Demographic and clinical characteristics, including sex, age, body mass index, mean arterial pressure, heart rate, hypertension, hyperlipidemia, and smoking status, were similar among the iRSWA, iRBD, PD + RBD, and control groups, with no statistically significant differences. In contrast, SCOPA‐AUT (Figure 2) and RBDQ‐HK scores differed significantly among the four groups (Table 1) (*p* < 0.001).

**FIGURE 1:**
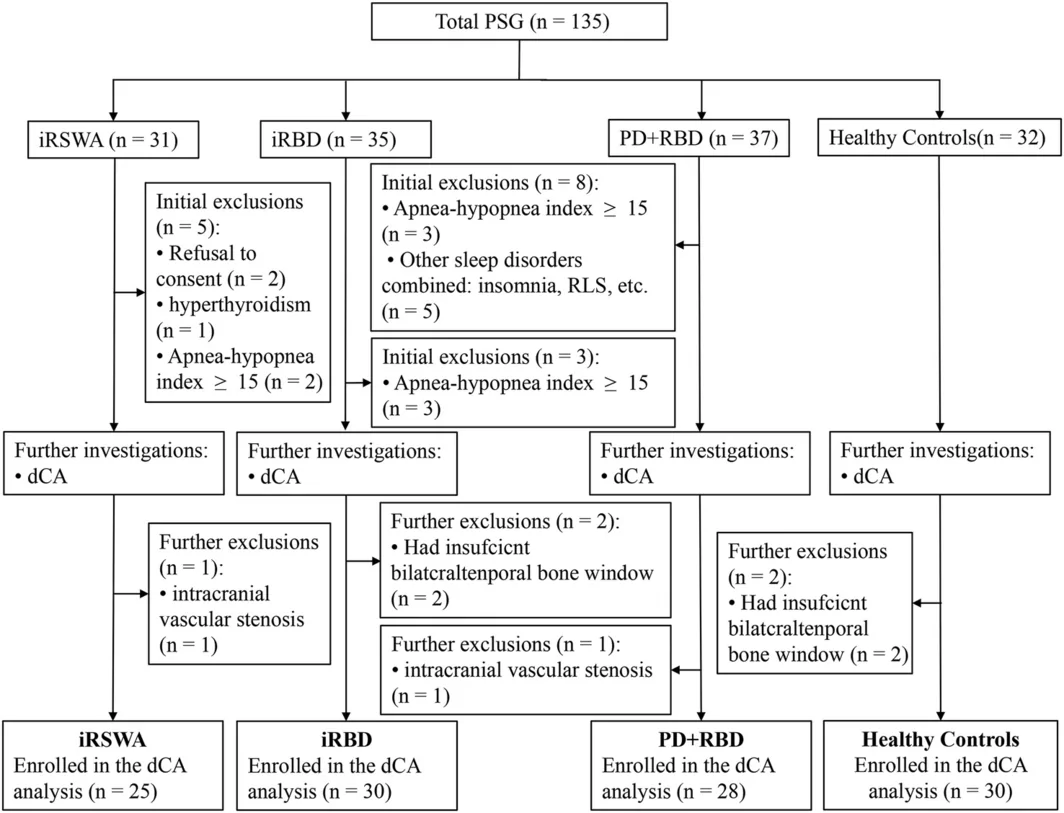
Flowchart shows the screening process for participants. dCA, dynamic cerebral autoregulation; iRBD, isolated rapid eye movement sleep behavior disorder; iRSWA, isolated rapid eye movement sleep without atonia; PD, Parkinson disease; PSG, polysomnography.

**TABLE 1:** Clinical characteristics in iRSWA, iRBD, PD+RBD patients and controls.

	Controls (*n* = 30)	iRSWA (*n* = 25)	iRBD (*n* = 30)	PD+RBD (*n* = 28)	$\chi^{2}$/F/H	*P*
Male, *n* (%)	18 (60%)	15 (60%)	17 (56.7%)	13 (46.4%)	1.399	0.706
Age (years)	52.23 ± 8.55	51.96 ± 8.91	50.57 ± 8.71	52.36 ± 5.91	0.306	0.821
BMI (kg/m^2^)	24.05 ± 3.46	23.70 ± 3.51	22.75 ± 1.67	24.06 ± 2.29	1.424	0.240
MAP (mmHg)	89.30 ± 13.90	89.84 ± 8.79	89.10 ± 9.70	89.68 ± 9.59	0.028	0.994
Heart rate (beats/min)	74.57 ± 8.11	73.04 ± 7.69	74.71 ± 10.06	74.82 ± 6.75	0.263	0.852
Hypertension, *n* (%)	6 (20%)	4 (16%)	4 (13.3%)	6 (21.4%)	0.818	0.845
Hyperlipidemia, *n* (%)	3 (10%)	3 (12%)	4 (13.3%)	3 (10.7%)	0.188	0.979
Smoking, n (%)	6 (20%)	6 (24%)	6 (20%)	5 (17.9%)	0.317	0.957
SCOPA‐AUT, total	2.37 ± 1.10^a^	7.40 ± 3.56^b^	12.23 ± 5.57^c^	16.32 ± 5.84^d^	53.251	< 0.001*
Gastrointestinal	0.50 (0.00, 1.00)^a^	3.00 (2.00, 5.50)^b^	3.00 (2.00, 6.00)^b^	5.00 (3.00, 6.75)^b^	48.340	< 0.001*
Urinary	1.00 (0.00, 2.00)^a^	2.00 (1.00, 3.00)^a^	4.00 (2.75, 7.00)^b^	7.50 (4.25, 10.75)^b^	60.388	< 0.001*
Cardiovascular	0.00 (0.00, 2.00)^a^	1.00 (0.00, 2.00)^a^	2.00 (0.75, 3.00)^b^	2.00 (1.00, 3.00)^b^	22.912	< 0.001*
Thermoregulatory	0.00 (0.00, 0.00)^a^	0.00 (0.00, 1.00)^b^	0.00 (0.00, 1.00)^b^	0.00 (0.00, 1.00)^b^	13.608	0.003*
Pupillomotor	0.00 (0.00, 0.00)^a^	0.00 (0.00, 0.00)^ab^	0.00 (0.00, 1.00)^b^	0.00 (0.00, 1.00)^b^	14.747	0.002*
Sexual	0.00 (0.00, 0.00)^a^	0.00 (0.00, 0.00)^ab^	0.00 (0.00, 1.00)^b^	0.00 (0.00, 1.00)^b^	20.161	< 0.001*
RBDQ‐HK	2.57 ± 1.98^a^	7.28 ± 3.75^a^	36.17 ± 13.92^b^	42.61 ± 16.08^c^	96.405	< 0.001*
End‐tidal CO_2_ (mmHg)	35.66 ± 4.74	36.01 ± 4.05	35.37 ± 5.02	36.40 ± 4.86	0.260	0.854

BMI: body mass index; iRBD: isolated rapid eye movements sleep behavior disorder; iRSWA: isolated rapid eye movements sleep without atonia; PD: Parkinson disease; MAP: mean arterial pressure; RBD‐HK: rapid eye movements sleep behavior disorder questionnaire‐Hong Kong; SCOPA‐AUT: scales for outcomes in Parkinson’s disease‐autonomic; *P value < 0.05 (statistically different). Similar superscript letters indicate no significant difference, while different superscript letters indicate significant difference in Bonferroni’s post‐hoc test and Kruskal‐Wallis Test.

**FIGURE 2:**
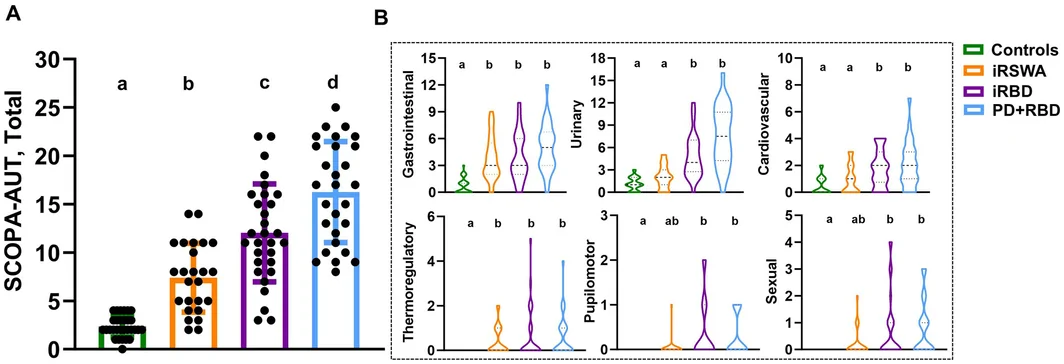
Comparison of the SCOPA‐AUT scale for all groups. Similar lowercase letters indicate no significant difference, while different lowercase letters indicate significant difference (*p* < 0.05) (Bonferroni's post hoc test). SCOPA‐AUT, scales for outcomes in Parkinson's disease‐autonomic.

### 3.2 Correlation Between REM Sleep Amounts and RSWA

REM sleep percentage was significantly lower in the iRBD and PD + RBD groups than in controls (Table 2). Across all patients, REM sleep percentage showed a nonsignificant trend toward a negative correlation with phasic RSWA (*r* =−0.2083, *p* = 0.0588), whereas no significant correlation was observed with tonic RSWA (*r* =−0.1547, *p* = 0.1626) (Figure 3).

**TABLE 2:** Polysomnography parameters in iRSWA, iRBD, PD+RBD patients and controls.

	Controls (*n* = 30)	iRSWA (*n* = 25)	iRBD (*n* = 30)	PD+RBD (*n* = 28)	*F*	*P*
Total sleep time (min)	375.48 ± 38.08	368.03 ± 78.37	347.41 ± 61.31	345.82 ± 56.91	1.796	0.152
Sleep onset latency (min)	15.53 ± 3.03^a^	15.89 ± 3.41^a^	18.25 ± 3.46 ^a^	28.14 ± 8.29^b^	38.762	< 0.001*
REM sleep latency (min)	98.33 ± 34.45^ab^	89.83 ± 26.28 ^a^	118.64 ± 55.20^b^	115.54 ± 28.19^ab^	3.534	0.017*
Sleep efficiency (%)	88.45 ± 7.32^a^	84.35 ± 11.50^ab^	77.58 ± 10.74^b^	69.31 ± 11.14^c^	19.065	< 0.001*
Stage N1 (%)	4.42 ± 1.24^a^	5.94 ± 5.02^a^	16.22 ± 7.97^b^	20.47 ± 9.18^b^	39.510	< 0.001*
Stage N2 (%)	50.47 ± 6.97	52.85 ± 8.44	54.90 ± 7.10	51.54 ± 10.54	1.553	0.205
Stage N3 (%)	21.20 ± 5.02	20.40 ± 5.49	18.66 ± 5.84	18.60 ± 3.94	1.849	0.143
REM sleep (%)	23.90 ± 8.59^a^	20.80 ± 6.65^a^	10.22 ± 7.99^b^	8.95 ± 7.67^b^	26.496	< 0.001*
PLMI (events/h)	0.89 ± 0.77^a^	12.78 ± 11.08^b^	19.05 ± 6.19^c^	18.53 ± 9.54^c^	35.325	< 0.001*
Arousal index (events/h)	6.00 ± 3.58^a^	21.16 ± 5.03^b^	11.67 ± 9.62^c^	13.61 ± 8.40^c^	20.806	< 0.001*
AHI (events/h)	2.12 ± 1.61	2.41 ± 1.36	2.66 ± 1.28	2.55 ± 1.37	0.810	0.491
Total RSWA (%)	—	33.50 ± 4.81^a^	40.05 ± 8.80^b^	41.36 ± 8.02^b^	8.165	0.001*
Tonic RSWA (%)	—	19.24 ± 5.17	20.44 ± 4.13	19.74 ± 4.29	0.494	0.612
Phasic RSWA (%)	—	14.25 ± 3.08^a^	20.16 ± 7.61^b^	21.61 ± 7.77^b^	8.964	< 0.001*

AHI: apnea‐hypopnea index; PLMI: periodic limb movement index; REM: rapid eye movement; RSWA: REM sleep without atonia; Stage N1: stage 1 non‐REM; Stage N2: stage 2 non‐REM; Stage N3: stage 3 non‐REM. *P value < 0.05 (statistically different). Similar superscript letters indicate no significant difference, while different superscript letters indicate significant difference in Bonferroni’s post‐hoc test.

**FIGURE 3:**
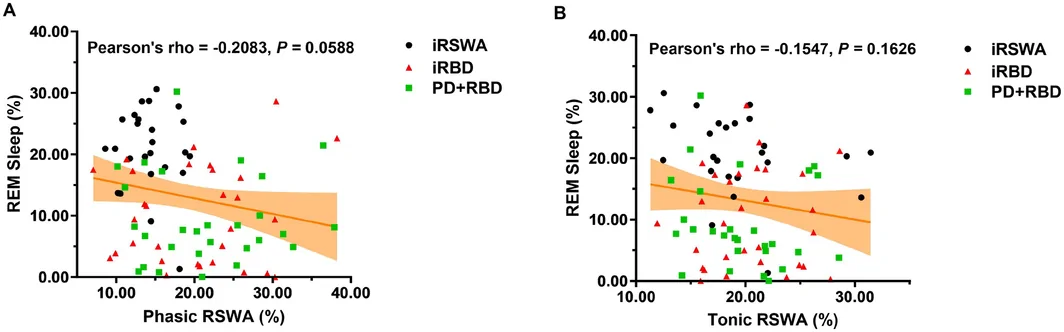
Relations between REM sleep and phasic RSWA (A) or tonic RSWA (B). REM, rapid eye movement; RSWA, rapid eye movement sleep without atonia.

### 3.3 Comparison of dCA Parameters

No significant differences were observed between the right and left sides for any dCA parameters, including phase difference, gain, and RoRc. Therefore, bilateral mean values were used for subsequent analyses. Phase difference was significantly lower in the iRSWA (45.81° ± 10.78°), iRBD (38.62° ± 10.03°), and PD + RBD (39.61° ± 9.43°) than in the control group (54.39° ± 9.59°, *p* < 0.001) (Figure 4).

**FIGURE 4:**
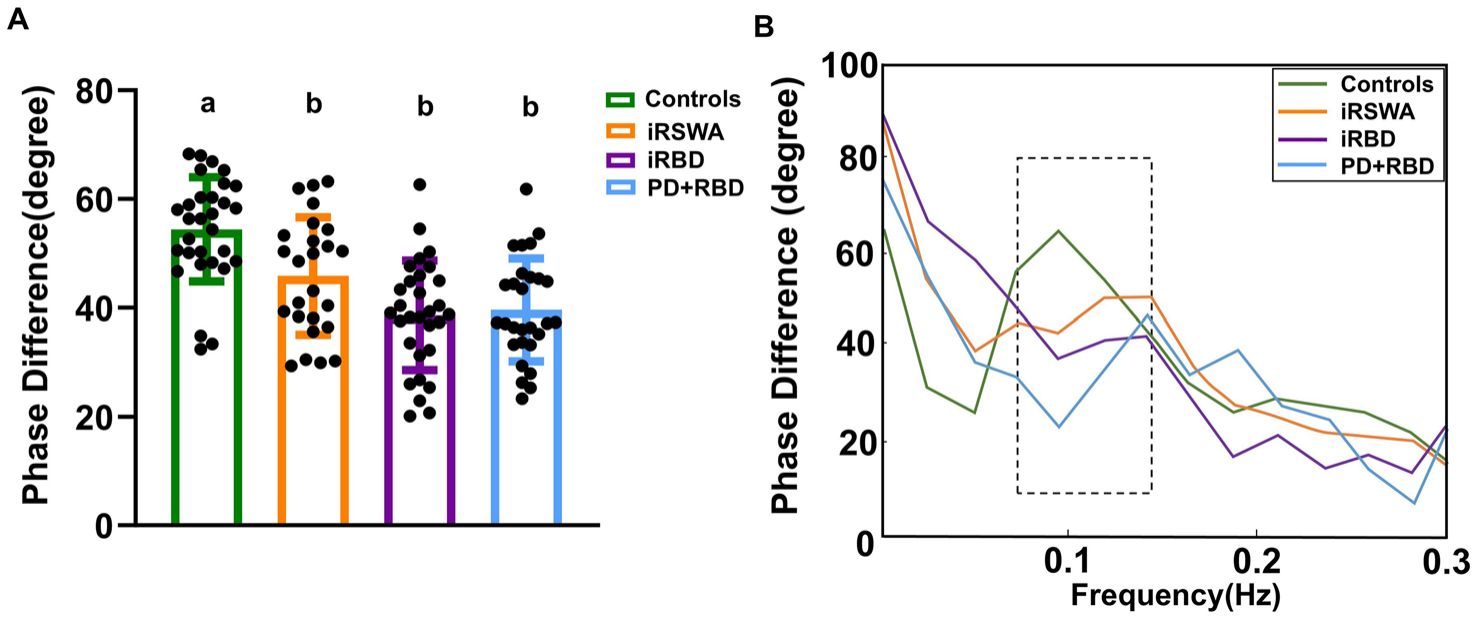
The dynamic cerebral autoregulation parameter and statistical distributions in each group. Statistical distributions of average phase difference (A) and its transfer function (B) in each group. Similar lowercase letters indicate no significant difference, while different lowercase letters indicate significant difference (*p* < 0.05) (Bonferroni's post hoc test). Dashed frame represents specific frequency domain (0.06–0.12 Hz).

### 3.4 Correlation Analysis for dCA Parameters With RSWA and REM Sleep Amounts

Pearson correlation analysis showed that phase difference was significantly correlated with phasic RSWA in the iRSWA (*r* = −0.5342, *p* = 0.006), iRBD (*r* = −0.4546, *p* = 0.0116), and PD + RBD (*r* = −0.3989, *p* = 0.0355). Across all patients, phase difference was also significantly correlated with phasic RSWA (*r* =‐0.4788, *p* < 0.001). Across all participants, phase difference was significantly correlated with  REM sleep percentage (*r* = 0.3629, *p* < 0.001) (Figure 5).

**FIGURE 5:**
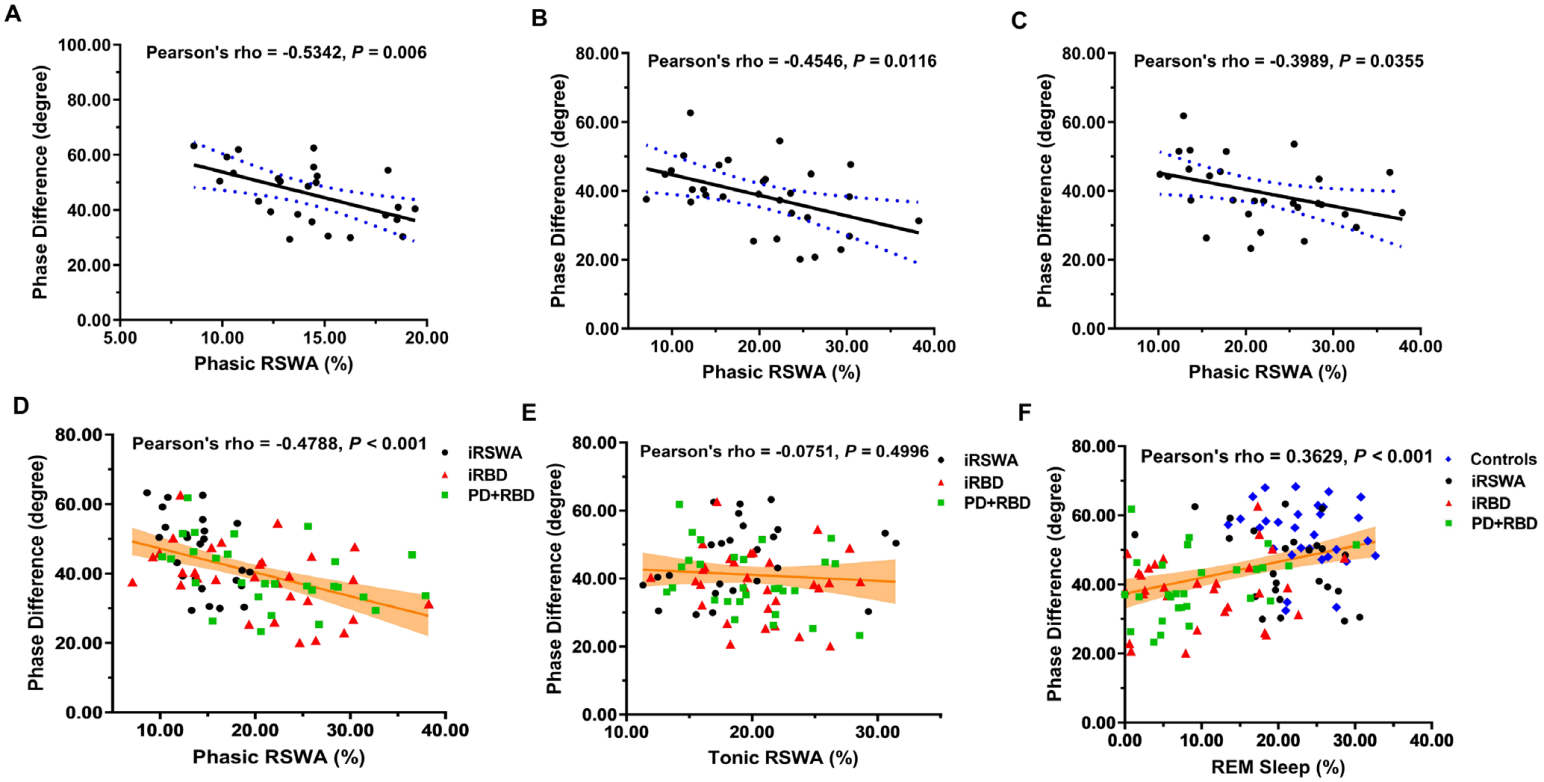
Relations between phase difference and phasic RSWA in iRSWA group (A), iRBD group (B), and PD + RBD group (C). Relations between phase difference and phasic RSWA (D), tonic RSWA (E), and REM sleep (F) in all subjects. iRBD, Isolated rapid eye movement sleep behavior disorder; iRSWA, isolated rapid eye movement sleep without atonia; PD, Parkinson disease; REM, rapid eye movement.

### 3.5 Univariable and Multivariable Analyses of Factors Associated With dCA

Table 3 presented the univariable and multivariable regression analyses of phase difference. In the univariate analysis, phase difference was significantly associated with stage N1 (*p* = 0.047), stage REM (*p* = 0.042), arousal index (*p* = 0.012), PLMI (*p* = 0.048), total RSWA (*p* < 0.001), and phasic RSWA (*p* < 0.001). Variables with *p* < 0.10 in the univariable analysis, including SCOPA‐AUT, stage N1, stage REM, arousal index, PLMI, total RSWA, and phasic RSWA, were entered into the multivariable model. After adjustment, none of these variables remained independently associated with phase difference.

**TABLE 3:** Univariable and multivariable analysis for the phase difference.

Factors	phase difference, degree			
	Univariable analysis		Multivariable analysis	
	β	*P*	β	*P*
Gender	0.040	0.719		
Age	0.093	0.404		
BMI	−0.100	0.370		
MAP	−0.102	0.357		
Heart Rate	−0.033	0.769		
Hypertension	0.014	0.900		
Hyperlipidemia	−0.023	0.838		
Smoking	0.017	0.879		
SCOPA‐AUT, total	−0.202	0.067^a^	−0.038	0.726
RBD‐HK	−0.095	0.395		
End‐tidal CO_2_ (mmHg)	−0.117	0.290		
Total sleep time (min)	0.001	0.992		
Sleep onset latency (min)	−0.061	0.587		
REM sleep latency (min)	−0.180	0.103		
Sleep efficiency (%)	0.043	0.698		
Stage N1 (%)	−0.218	0.047^ab^	−0.062	0.597
Stage N2 (%)	0.044	0.692		
Stage N3 (%)	−0.024	0.832		
REM Sleep (%)	0.223	0.042^ab^	0.028	0.815
Arousal Index (events/h)	0.275	0.012^ab^	0.153	0.147
AHI (events/h)	0.129	0.245		
PLMI (events/h)	−0.218	0.048^ab^	−0.026	0.815
Total RSWA (%)	−0.463	<0.001^ab^	−0.169	0.367
Tonic RSWA (%)	−0.075	0.500		
Phasic RSWA (%)	−0.479	<0.001^ab^	−0.268	0.165

AHI: apnea‐hypopnea index; BMI: body mass index; MAP: mean arterial pressure; PLMI: periodic limb movement index; RBD‐HK: rapid eye movements sleep behavior disorder questionnaire‐Hong Kong; REM: rapid eye movement; RSWA: REM sleep without atonia; SCOPA‐AUT: scales for outcomes in Parkinson’s disease‐autonomic; Stage N1: stage 1 non‐REM; Stage N2: stage 2 non‐REM; Stage N3: stage 3 non‐REM. ^a^Nominally significant values (*P* < 0.1) included in the multivariable model; ^b^ *P* value < 0.05 (statistically different).

## 4 Discussion

The current study demonstrated varying degrees of autonomic dysfunction across patients with iRSWA, iRBD, and PD + RBD. DCA was impaired across these patient groups and was significantly associated with REM sleep duration and phasic RSWA measured by PSG. These findings may reflect functional alterations in central autonomic and cerebrovascular regulation across the RBD spectrum.

### 4.1 dCA Impairment Across the RBD Spectrum

To the best of our knowledge, this study is the first to demonstrate compromised dCA in patients with iRSWA and to directly compare these alterations with those observed in iRBD and PD + RBD. Previous studies have suggested that iRSWA may represent a prodromal stage of RBD [3, 28]. Barone et al. analyzed electrocardiographic data obtained from PSG in individuals with iRSWA during continuous 5‐min arousal‐free periods and reported reduced heart rate variability, suggesting autonomic dysfunction in this population [29]. Consistent with these findings, our results showed a greater burden of autonomic symptoms in the patients with iRSWA than healthy controls, as assessed by the SCOPA‐AUT, particularly in the gastrointestinal and thermoregulatory domains. Moreover, phase difference was reduced in patients with iRSWA and was intermediate between that observed in healthy controls and patients with iRBD, suggesting an intermediate degree of dCA impairment.

The persistent deterioration of skeletal muscle atonia during REM sleep in patients with iRSWA has also been proposed as a potential early marker of α‐synucleinopathy‐related neurodegeneration [30]. Taken together, these findings are consistent with the possibility that iRSWA may represent an early stage within the RBD spectrum, although it may remain stable and non‐progressive in some individuals. These findings also support the inclusion of standardized autonomic and cerebrovascular assessments in longitudinal cohorts, such as the DREAMER study, to improve phenotypic characterization and facilitate the identification of markers associated with phenoconversion [31].

Previous studies have demonstrated widespread and regionally heterogeneous cerebral perfusion abnormalities in patients with iRBD. Early work reported reduced perfusion in the pons and upper frontal regions [16]. Mazza et al. [32] subsequently observed increased perfusion in the hippocampus, putamen, and pons, together with decreased perfusion in the frontal, temporal, and parietal lobes [32]. Hanyu et al. [33] further reported reduced regional cerebral blood flow in the parieto‐occipital cortex, limbic regions, and cerebellar hemispheres, supporting the concept that iRBD may represent a presymptomatic stage of α‐synucleinopathies, as similar perfusion abnormalities have been reported in established neurodegenerative diseases. Longitudinal studies have also demonstrated progressive reductions in regional cerebral blood flow in the bilateral temporo‐parieto‐occipital cortices, particularly in the right posterior cingulate cortex [34]. Although these studies reported heterogeneous perfusion patterns, they collectively indicate that cerebral perfusion is altered in patients with iRBD. In this study, dCA was significantly impaired in patients with iRBD, providing complementary evidence of altered cerebrovascular regulation in this population. Autonomic dysfunction is also a well‐recognized feature of iRBD. Baldelli et al. [35] reported that cardiovascular autonomic failure was associated with an increased risk of neurodegenerative phenoconversion in patients with iRBD. Other studies have similarly suggested that greater autonomic impairment may be associated with a higher risk and more rapid phenoconversion to clinically manifest neurodegenerative disease [36, 37]. Taken together, these findings suggest that autonomic and cerebrovascular dysregulation may coexist in iRBD, although their causal relationship and potential role in neurodegenerative progression remain to be established.

Previous findings regarding cerebral autoregulation in patients with PD have been inconsistent, and the specific contribution of concomitant RBD remains insufficiently characterized. Importantly, most previous studies in PD did not distinguish between patients with and without RBD. Angeli et al. [38] reported that PD patients without orthostatic intolerance exhibited only mild hypotensive responses to orthostatic stress, accompanied by compensatory intracranial vasodilation, suggesting relatively preserved cerebral autoregulation in this subgroup. Similarly, Niehaus et al. [39] found that, although autonomic circulatory control was impaired in PD patients without orthostatic dysregulation, cerebral hemodynamic regulation during orthostatic challenge remained largely preserved. In contrast, other studies have reported abnormal cerebral hemodynamic responses in PD. Vokatch et al. [40] observed prolonged latency of vasoconstrictive responses, suggesting impaired arteriolar vascular regulation, whereas Tsai et al. [41] demonstrated blunted cerebral hemodynamic responses to the cold pressor test, indicating altered cerebrovascular reactivity. These heterogeneous findings may partly reflect differences in study populations, disease stages, and the presence or absence of RBD. In this study, phase difference was significantly reduced in patients with PD + RBD, indicating impaired dCA in this subgroup. According to Braak's staging hypothesis, the ascending pathology of PD originates in the lower brainstem, with Stage I pathology involving the medulla and pons, and subsequently progressing to the midbrain and beyond in Stages II and III [42]. This pathological progression suggests that pontine structures, including the subcoeruleus (SC) region in humans, may be progressively affected across the spectrum from iRSWA to iRBD and PD with RBD. Animal studies provide additional mechanistic context: partial lesions of the sublaterodorsal nucleus (SLD), the corresponding REM‐atonia‐related region in rodents, can produce REM sleep and RBD‐like behaviors together with reduced and fragmented REM sleep, whereas more extensive SLD lesions can markedly suppress REM sleep and RBD‐like behaviors [14, 43]. In our cohort, REM sleep was also reduced across the iRSWA, iRBD, and PD + RBD groups. However, the relationship between SC dysfunction, REM sleep loss, and RSWA is likely nonlinear. Although phasic RSWA may reflect dysfunction within REM‐related motor control circuits, severe disruption of these circuits may reduce REM sleep itself, limiting a simple correlation between RSWA severity and the extent of SC pathology.

### 4.2 Potential Mechanisms Linking REM Sleep, Autonomic Dysfunction, and dCA

Many factors influence dCA, with the autonomic nervous system serving as a major regulator. During REM sleep, autonomic activity becomes markedly variable and is accompanied by increased cerebral blood flow compared with wakefulness and NREM sleep [44]. Disturbances of REM sleep process, as observed in RBD and sleep apnea, may therefore affect blood pressure regulation, cerebral perfusion, and ultimately dCA [45]. Our study revealed significant variations in SCOPA‐AUT scores across multiple domains, including gastrointestinal, urinary, cardiovascular, thermoregulatory, pupillomotor, and sexual dysfunction, among the iRSWA, iRBD, PD + RBD, and control groups. In addition, phasic, but not tonic RSWA was correlated with phase difference, suggesting that phasic REM sleep mechanisms may be particularly relevant to autonomic and cerebrovascular regulation. Experimental studies suggest that REM sleep involves partially distinct phasic and tonic neural mechanisms [46, 47]. Phasic generators are responsible for phasic eye movements, penile erection, phasic respiration, cardiovascular activation, and inhibitory projections to spinal motor neurons that suppress postural muscle tone. In contrast, tonic generators suppress tonic muscle activity, respiration and heart rate. Previous studies suggest that tonic and phasic RSWA may reflect dysfunction of partially distinct neural mechanisms involved in REM sleep motor control [48, 49]. The increased tonic and phasic RSWA observed in our study may be consistent with altered REM sleep motor regulation, although the underlying neural mechanisms cannot be determined from the present data. Consistent with this concept, reduced phasic eye movements and attenuated autonomic fluctuations during REM sleep have been reported in patients with RBD [50, 51].

The precise role of the SC region in autonomic regulation remains incompletely understood. It has been shown that blood pressure increases during REM sleep depend on the forebrain as midbrain transection abolishes blood pressure increases during REM sleep in cats [52]. It has therefore been proposed that REM‐related brainstem circuits may interact with hypothalamic autonomic centers, including the paraventricular nucleus, which participates in cardiovascular and cerebrovascular regulation [52, 53]. Alternatively, dCA impairment in RBD may also reflect more widespread brainstem autonomic involvement associated with α‐synuclein pathology. According to Braak staging, early medullary and pontine pathology may involve autonomic brainstem circuits, including the A1 noradrenergic cell group, potentially contributing to impaired cerebrovascular regulation.

Furthermore, REM sleep regulation involves multiple neurotransmitter systems, including serotonergic neurons in the dorsal raphe nucleus, noradrenergic neurons in the locus coeruleus, and cholinergic neurons in the pontine nucleus. Disruptions of REM sleep may therefore be accompanied by alterations in serotonergic, noradrenergic, cholinergic, and other neurochemical systems [54, 55]. Norepinephrine is involved in both sleep‐autonomic regulation and cerebrovascular control, whereas 5‐hydroxytryptamine is a potent vasoactive neurotransmitter that may also influence dCA [56].

### 4.3 Sleep Abnormalities and Disease Progression

Sleep architecture differed significantly among the groups, along with differences in PLMI, arousal index, and phasic RSWA. However, none of these sleep‐related variables remained independently associated with phase difference in the multivariable analysis. The abnormalities observed in the iRSWA group may support its proposed position within the early RBD spectrum, although iRSWA may remain stable and non‐progressive in some individuals. Overall, these findings may reflect partially overlapping pathophysiological features across the RBD spectrum. Longitudinal studies are needed to clarify the natural history of iRSWA and to determine whether specific individuals are at increased risk of progression to clinically manifest RBD or other α‐synucleinopathy‐related neurodegenerative diseases.

Although REM sleep percentage was significantly reduced in the iRBD and PD + RBD groups, this finding should be interpreted cautiously. Previous human polysomnographic studies have reported no significant differences in REM sleep percentage or duration between patients with iRBD and controls [57, 58]. In our cohort, the reduced REM sleep percentage was accompanied by lower sleep efficiency and increased stage N1 sleep, suggesting that it may reflect broader sleep fragmentation or altered sleep architecture rather than a specific effect of RBD on REM sleep. In addition, RSWA may complicate REM sleep identification in RBD [59]. Although sleep staging was performed according to standard criteria by experienced scorers, a minor influence of RSWA on REM sleep estimation cannot be completely excluded.

## 5 Limitations

Our study had some limitations. First, the clinical history of iRBD symptoms was obtained from patients and their bed partners, which may have introduced recall bias despite confirmation by v‐PSG. Second, unsuccessful temporal bone window insonation in some elderly participants may have restricted their inclusion. Third, the exclusion of participants with intracranial or extracranial vascular stenosis or occlusion, insomnia, or restless legs syndrome, although intended to minimize potential confounding effects on cerebral hemodynamics, sleep continuity, and autonomic activity, may have introduced selection bias and limited the generalizability of our findings. Further longitudinal studies with larger and more representative samples are warranted to validate and extend these findings.

## 6 Conclusion

This study demonstrated that dCA was impaired in patients with iRSWA, iRBD, and PD + RBD, which was correlated with REM sleep amount and phasic RSWA on PSG. Further studies are needed to clarify the underlying mechanisms, determine the relationship of RSWA and RBD with neurodegenerative progression, and evaluate the clinical significance and potential implications of these findings.

## Author Contributions

**Yanan Zhang:** data curation, methodology, software, writing – original draft; **Ying Piao:** data curation, methodology, investigation; **Qingqing Sun:** data curation, formal analysis, methodology; **Huimin Li:** conceptualization, methodology, supervision; **Yaru Wang:** data curation, formal analysis, methodology. **Jun Lu:** conceptualization, methodology, supervision; **Zhenni Guo:** conceptualization, methodology, supervision. **Zan Wang:** funding acquisition, methodology, supervision, writing – review and editing.

## Funding

The article was supported by the Brain Science and Brain‐like Intelligence Technology–National Science and Technology Major Project (Grant number 2021ZD0204303) and the National Key R&D Program of China (Grant number 2022YFC2503904) to Z.W.

## Ethics Statement

The study was approved by the Ethics Committee of the First Hospital of Jilin University (Approval No: 2016‐294) and followed the guidelines of the Declaration of Helsinki (1964). All participants or their relevant guardians provided written informed consent.

## Conflicts of Interest

The authors declare no conflicts of interest.

## Data Availability

The raw data supporting the conclusions of this article will be made available from the corresponding authors upon reasonable request.
